# Chemotherapy-Induced Amenorrhea and Its Prognostic Significance in Premenopausal Women With Breast Cancer: An Updated Meta-Analysis

**DOI:** 10.3389/fonc.2022.859974

**Published:** 2022-04-05

**Authors:** Yifei Wang, Yaming Li, Jingshu Liang, Nan Zhang, Qifeng Yang

**Affiliations:** ^1^ Department of Breast Surgery, General Surgery, Qilu Hospital of Shandong University, Jinan, China; ^2^ Breast Cancer Center, Jinan Central Hospital, Shandong First Medical University, Jinan, China; ^3^ Pathology Tissue Bank, Qilu Hospital of Shandong University, Jinan, China; ^4^ Research Institute of Breast Cancer, Shandong University, Jinan, China

**Keywords:** breast cancer, premenopausal, chemotherapy-induced amenorrhea, prognosis, meta-analysis

## Abstract

**Objective:**

Chemotherapy-induced amenorrhea (CIA) is one of the most common side effects in premenopausal patients with breast cancer, and several factors may contribute to the incidence of CIA. In this meta-analysis, we aimed to summarize clinical risk factors associated with CIA incidence and to evaluate their prognostic effects in patients with breast cancer.

**Methods:**

Three electronic databases (Cochrane Library, EMBASE, and MEDLINE) were systematically searched for articles published up to October 2021. The articles included clinical trials that evaluated risk factors associated with CIA and their prognostic value in treatment. For the meta-analysis, pooled odds ratio estimates (ORs) and 95% confidence intervals (CIs) were calculated using the inverse variance-weighted approach, in addition to publication bias and the chi-square test.

**Results:**

A total of 68 studies involving 26,585 patients with breast cancer were included in this meta-analysis, and 16,927 patients developed CIA. From the 68 studies, 7 risk factors were included such as age group, hormone receptor (HR) status, estrogen receptor (ER) status, progesterone receptor (PR) status, tamoxifen administration, chemotherapeutic regimen, and tumor stage. Based on our results, patients with age of ≤40, HR-negative status, ER-negative status, PR-negative status, no use of tamoxifen, and use of anthracycline-based regimen (A) compared with anthracycline-taxane-based regimen (A+T) were associated with less incidence of CIA in patients with breast cancer. Moreover, CIA was associated with favorable disease-free survival (OR = 0.595, 95% CI = 0.537 to 0.658, p < 0.001) and overall survival (OR = 0.547, 95% CI = 0.454–0.660, *p* < 0.001) in premenopausal patients with breast cancer.

**Conclusion:**

Age, HR status, ER status, PR status, tamoxifen administration, and chemotherapeutic regimen can be considered independent factors to predict the occurrence of CIA. CIA is a favorable prognostic factor in premenopausal patients with breast cancer. CIA should be a trade-off in the clinical management of premenopausal patients with breast cancer, and further large cohort studies are necessary to confirm these results.

## Introduction

Breast cancer has surpassed lung cancer to become the most frequently diagnosed cancer among women worldwide as per the latest data released by the International Agency for Research on Cancer of the World Health Organization in 2020 (1). With the development of the pharmaceutical field, the comprehensive treatment of breast cancer is constantly updated and the current systemic treatment includes surgery, chemotherapy, radiotherapy, endocrine therapy, and target therapy (2). Chemotherapy is still a predominant adjuvant therapy for the treatment of breast cancer, which could effectively prolong patient survival and reduce the recurrence rate of cancer. However, patients may develop various side effects including myelosuppression, cardiotoxicity, ovarian failure, nausea, and diarrhea, which affect the quality of life (3, 4). The early prevention and treatment of complications caused by chemotherapy have become an important supplement in the chemotherapy strategy for breast cancer.

Chemotherapy-induced amenorrhea (CIA) is a common complication observed in premenopausal women with breast cancer, and the incidence of CIA ranges from 15% to 94% (5) in patients with breast cancer after receiving chemotherapy. CIA is caused by suppression of ovarian function, which can lead to genitourinary dysfunctions, infertility, and peri-menopausal symptoms such as hot flushes and sweats. Furthermore, long-time hormone deprivation can increase osteoporosis and cardiovascular risk, thus causing both physical and psychological distress among patients (6–8). Moreover, published data indicate that the major concern for premenopausal women receiving chemotherapy for breast cancer is to preserve their future childbearing potential (9). Therefore, it is of great value to identify individuals who are vulnerable to CIA, to identify risk factors, and to determine their prognostic value for treatments of patients with breast cancer.

Although the definition of CIA varies among studies, risk factors identified for CIA include age (10), hormone receptor (HR) status (11, 12), tamoxifen administration, and chemotherapeutic regimens (12–15). However, these studies have some limitations such as small sample size and the inclusion of single or few potential risk factors. Importantly, some risk factors are debatable. Parulekar et al. reported that the HR status showed no significant association with the incidence of CIA, which was 73.3% in the receptor-positive group and 74.0% in the receptor-negative group (11). On the contrary, Yoo reported that the HR-positive status is one of the risk factors of CIA, with 64.4% incidence in the HR-positive group and 42.7% incidence in the HR-negative group (12). The use of tamoxifen as a risk factor of CIA is also debatable (13, 15). The incidence of CIA is closely related to the adjuvant chemotherapeutic regimen and dosage (16). The most common clinically used chemotherapeutic regimens are anthracycline-based (A) and anthracycline-taxane-based (A+T) (17). The addition of taxane to the anthracycline regimen could improve the overall survival (OS) rate of patients compared with anthracycline alone (18). However, there is no consensus on the effect of the chemotherapeutic regimen on CIA incidence. Some studies have reported that A+T could significantly increase the occurrence rate of CIA (14), whereas others reported that the incidence of CIA and the use of different regimens are not correlated (13, 19). As amenorrhea would impair the quality of life in premenopausal patients with breast cancer (20), studying risk factors for their prognostic effects on CIA incidence is necessary. Consistent findings are not available based on previous studies (21, 22). Walshe et al. reviewed 23 studies, and 10 of them demonstrated survival benefits of CIA (23). Thus, further confirmation of risk factors associated with CIA and their prognostic value are warranted.

In this study, we aimed to perform an updated meta-analysis to achieve more reliable and comprehensive data on specific risk factors associated with the CIA. Moreover, we aimed to determine their exact prognostic value for CIA among premenopausal women with breast cancer receiving adjuvant chemotherapy.

## Methods

### Search Strategy

Three electronic databases (Cochrane Library, EMBASE, and MEDLINE) were quarried with the inclusion dates between January 1900 and October 2021, and specific keywords and free-text searches were used in the following combinations: amenorrhea, breast cancer, breast neoplasm, chemotherapy, ovarian toxicity, and CIA. We also used the “related articles” function to broaden the search and manually searched the reference lists of the retrieved literature to identify the relevant literature. Copies of all eligible studies were collected and read. In case of overlap in the patient cohorts across more than one study, only data from the most recent publication were utilized. The studies and databases performed without language or region restrictions were included in our meta-analysis.

### Inclusion and Exclusion Criteria

Papers included in our meta-analysis met all of the following inclusion criteria: (a) studies on breast cancer patients in the premenopausal age who received chemotherapy; (b) papers in which one or more factors associated with the incidence of CIA were discussed; (c) at least 20 patients were enrolled; (d) the study had to be published after 1990; and (e) in case of studies including patients both with and without the addition of GnRH analogues, we only extracted the data without the addition of GnRH analogues. The major exclusion criteria were as follows: (a) not meeting the inclusion criteria; (b) papers with insufficient data; and (c) the category of the paper was not an editorial, letter, review article, case report, or animal experimental study.

### Data Abstraction and Quality Assessment

Based on the inclusion and exclusion criteria above, the following data parameters were extracted for each study: the name of the first author, year of publication, the total number of patients analyzed, patient characteristics, country of origin for the study, definition of CIA, the incidence of CIA based on different risk factors, the 5-year disease-free survival (DFS), and the overall survival (OS), if mentioned. Information was carefully and independently extracted from all eligible publications by two of the authors, and any disagreement between the researchers was resolved by discussions until reaching a consensus. In case of failing to reach a consensus, a third investigator (an experienced professional breast surgeon) was consulted to resolve the dispute. The quality of observational studies was assessed using the Newcastle–Ottawa quality assessment tool (24). The Cochrane Risk of Bias Tool was used to assess the quality of the randomized control trials (RCTs) (25). A score of 0–9 was allocated to each observational study. Observational studies achieving scoring 6–9 points were considered to be high quality, studies scoring 4–5 points were rated as moderate quality, and studies scoring 3 or fewer points were regarded as low quality.

### Statistical Analysis

Stata V.12 software was utilized for all statistical analyses. The outcomes, OR, and 95% confidence intervals (CIs) were calculated, and the association between different risk factors and the incidence of CIA as well as its prognostic effect were assessed. Pooled ORs and subgroup analysis were performed, with the application of the Z-test to determine its statistical significance. Subgroup analyses were further conducted for the varied definitions of CIA across studies. Statistical heterogeneity was calculated by the chi-square test,and a fixed-effect-model was used for I^2^ <50%, with a random-effect model for I^2^ ≥50%, and further checked by sensitivity analyses. Publication bias was calculated using Begg’s test. For all tests, a probability level <0.05 was considered to indicate statistical significance. All statistical tests were two-sided.

## Results

### Search Results

**Figure 1:**
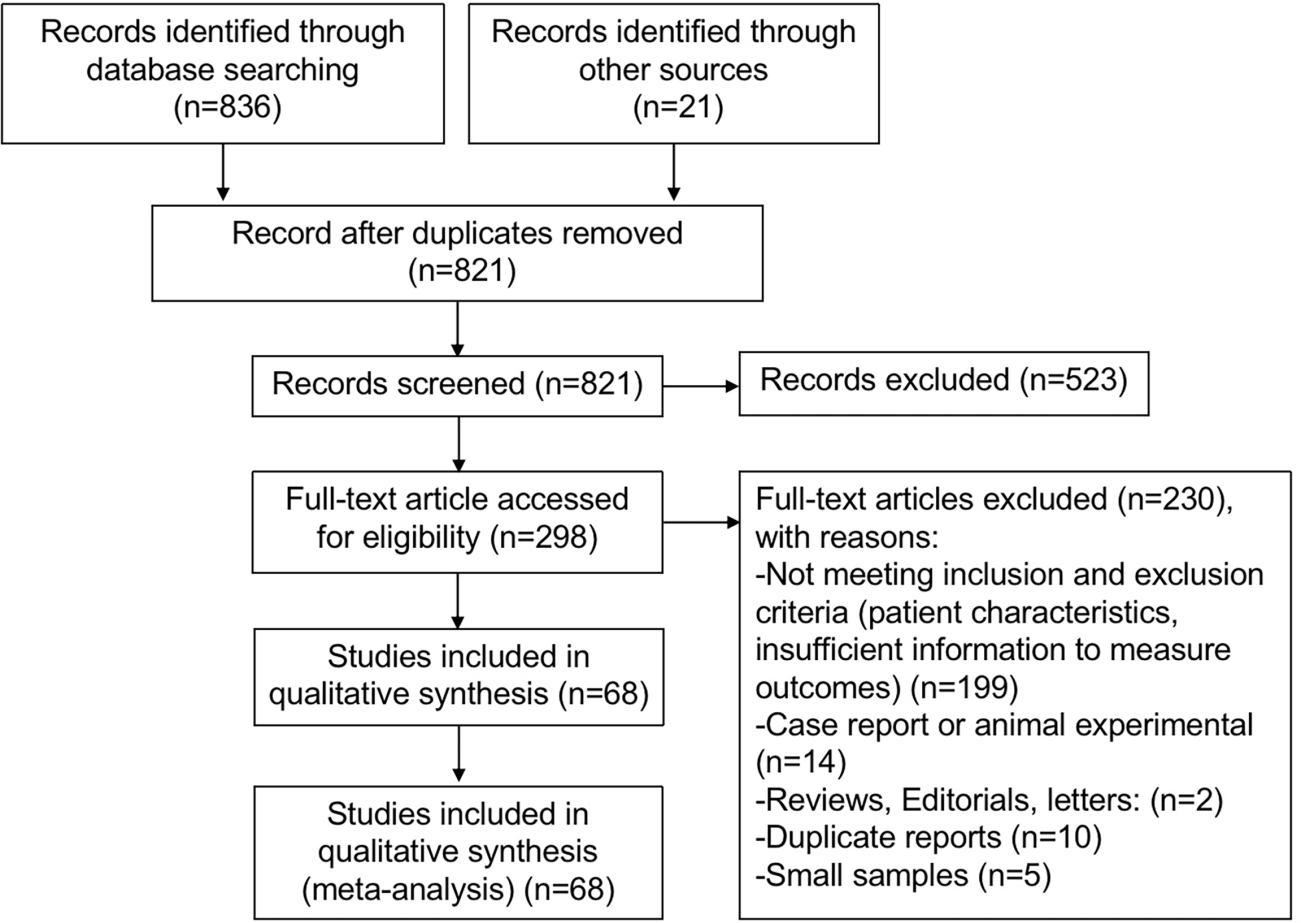
Flow diagram depicting the process of conducting meta-analysis.

Systematic retrieval using electronic searches of the Cochrane Library, EMBASE, and MEDLINE gave a total of 836 studies, and 21 other articles were identified from other sources by reviewing citations in the reference lists. After removing duplicates and reviewing titles and abstracts, a total of 298 articles were potentially eligible for inclusion. The full text of the 298 studies was reviewed thoroughly, and 230 articles were excluded because of (1) not meeting inclusion and exclusion criteria (patient characteristics, insufficient information to measure outcomes) (n = 199); (2) case report or animal experiments (n = 14); (3) reviews, editorials, and letters (n = 2); (4) duplicate reports (n = 10); and (5) small samples (n = 5). Ultimately, 68 studies (6, 10–15, 19, 20, 22, 26–83) met all inclusion criteria and were included in this meta-analysis. The flowchart of the literature search is shown in **Figure 1**.

### Characteristics of the Included Studies

The 68 filtered trials were published between January 1990 and October 2021, and the patient age ranged from 18 to 59 years, with sample sizes ranging from 45 to 2343. The definitions of CIA varied among the studies, 13 studies used 3 months as the minimal lasting time of amenorrhea, 19 studies used 6 months, and 20 studies used 12 months. Moreover, 17 studies did not explicitly state the definition of CIA. Of the total of 26,585 premenopausal patients with breast cancer who had received adjuvant chemotherapy, 16,927 (63.67%) patients developed CIA, with the CIA rate ranging from 15.06% to 98.33% among studies.

To evaluate risk factors associated with the occurrence of CIA, factors that were correlated with CIA among studies were identified. Finally, age (≤40 vs. >40), HR status (negative vs. positive), estrogen receptor (ER) status (negative vs. positive), progesterone receptor (PR) status (negative vs. positive), usage of tamoxifen (with vs. without), and chemotherapy regimens (anthracycline-based vs. anthracycline + Taxol based) were selected and pooled, with 43, 14, 9, 8, 27, and 22 studies enrolled in each analysis. The basic characteristics of patients with CIA in the 68 clinical trials and the associated prognostic factors are listed in **Table 1**.

**Table 1:** Basic characteristics of the 68 clinical trials studying patients with CIA.

Study	Years of study	Country	No. of patients	No. of CIA	Median age (y)	Definition of CIA	Parameter used	Study quality
Abdel-Razeq et al. (26)	2014–2017	Jordan	94	51	35.7	≥12 months	Age, CR	6
Abusief et al. (27)	1997–2005	USA	431	239	43	≥6 months	Age, TAM	7
Andersson et al. (28)	1982–1986	Denmark	634	444	——	No definition	TAM	8
Arslan et al. (29)	——*^a^*	USA	86	53	——	No definition	CR	4
Beex et al. (30)	1976–1987	Netherlands	77	47	——	No definition	PR, DFS, OS	5
Berliere et al. (31)	1997–2000	Belgian/French	154	98	43.5	≥12 months	CR	6
Bianco et al. (32)	1978–1989	Italy	221	166	43	≥3 months	Age, DFS	5
Boccardo et al. (33)	1983–1987	Italy	504	363	——	≥12 months	TAM	RCT
Bonadonna et al. (34)	1973	Italy	103	50	——	≥3 months	Age, DFS	RCT
Canney et al. (35)	2005	UK	1,333	639	——	No definition	Age	RCT
Davis et al. (13)	1998–2001	USA	159	78	42	≥12 months	Age, HR, TAM, CR	6
Di Cosimo et al. (36)	1993–2003	Italy	111	58	42	≥3 months	Age	6
Fornier, et al. (37)	1997–2003	USA	166	25	36	≥12 months	HR, TAM	7
Ganz, et al. (20)	1999–2004	Canada/USA	2,149	1403	——	≥6 months	HR, TAM	RCT
Goldhirsch et al. (38)	1981–1985	——	1,127	458	——	≥3 months	Age, HR, DFS	RCT
Goodwin et al. (39)	1992–1996	Canada	183	81	43.7	≥12 months	TAM	6
Han et al. (40)	2002–2005	Korea	285	144	40	≥3 months	Age, TAM, CR	8
IBCBG (41)	1978–1981	——	134	119	——	≥3 months	Age, TAM, DFS	RCT
IBCSG (42)	1993–1999	——	1,065	918	44	≥3 months	DFS	RCT
Jeon et al. (43)	2007–2013	Korea	249	128	64	≥6 months	Age, TAM, DFS	6
Jung et al. (44)	1995–2000	Korea	249	133	40	≥6 months	Age, TS, TAM, DFS, OS	8
Kim et al. (45)	2003–2006	Korea	324	261	40	≥3 months	Age, HR	6
Koga et al. (46)	2004–2009	Japan	101	97	45	No definition	Age	5
Lee et al. (47)	2000–2006	Korea	326	223	42	≥6 months	Age, HR	6
Li et al. (48)	2000–2005	China	160	107	42.86	≥3 months	Age, DFS	6
Liem et al. (49)	2008–2011	China	280	137	41	≥12 months	Age, ER, PR, CR	6
Lower et al. (50)	——	USA	109	50	——	No definition	Age, DFS, OS	5
Ludwig BCSG et al. (51)	1978–1981	Multinational	399	340	——	No definition	Age, ER, DFS	RCT
Martin et al. (52)	1997–1999	Canada	823	470	——	≥3 months	CR	RCT
Mehta et al. (53)	——	USA	70	54	——	No definition	Age	4
Meng et al. (19)	2007–2011	China	73	61	44	≥6 months	Age, CR	4
Najafi et al. (54)	1998–2008	Iran	226	154	40	≥6 months	Age, TS, ER, PR, TAM, CR	8
Narmadha et al. (55)	——	India	50	41	41	≥12 months	Age, CR	5
Okanami et al. (56)	2001–2005	Japan	66	48	37	No definition	HR, TAM, CR	5
Pagani et al. (57)	1986–1993	USA	1,196	736	——	≥3 months	Age, HR, ER, PR, DFS	RCT
Park et al. (58)	2001–2006	Korea	872	669	41	≥6 months	DFS	6
Parulekar et al. (11)	1989–1993	Canada	328	240	43.8	≥3 months	Age, HR	7
Perez-Fidalgo et al. (6)	1998–2005	Spain	305	237	44	≥12 months	Age, CR	8
Petrek et al. (59)	1998–2002	USA	523	268	——	No definition	CR	8
Poikonen et al. (22)	1990–1993	Finland	106	52	——	≥6 months	TS, HR, DFS, OS	7
Pourali et al. (60)	2001–2008	Iran	119	70	33.5	≥12 months	ER, PR, TAM, CR	5
Ravi et al. (61)	2017–2019	Pakistan	201	184	——	≥6 months	Age	RCT
Reh et al. (62)	2001–2005	USA	45	41	——	≥6 months	CR	4
Reimer et al. (63)	2001–2011	Germany	50	26	——	≥12 months	TS, ER, PR, TAM	6
Reyno et al. (64)	1984–1987	Canada	95	67	——	≥12 months	TAM, DFS, OS	RCT
Richards et al. (65)	1976–1985	UK	90	69	——	No definition	Age, DFS	RCT
Roche et al. (66)	1990–1998	France	169	104	44	≥6 months	Age	RCT
Rohutanda et al. (67)	2004–2008	Japan	60	59	——	No definition	CR	4
Rosendahl et al. (68)	——	Multinational	836	642	——	No definition	Age	RCT
Ruddy et al. (69)	2007–2010	——	64	18	44	≥12 months	Age, TAM	7
Ruddy et al. (70)	2005–2011	——	1,168	457	41	≥12 months	Age, ER, PR, TAM	RCT
Ruddy et al. (71)	2013–2016	——	76	23	——	≥6 months	ER, TAM	RCT
Sukumvanich et al. (14) *^b^*	1998–2002	USA	439	178	39	≥6 months	Age, CR	8
Sukumvanich et al. (14)	1998–2002	USA	445	128	——	≥12 months	Age, CR	8
Sverrisdottir et al. (72)	1990–1994	Multinational	52	32	——	No definition	TAM	RCT
Swain et al. (10)	1999–2004	USA	1,885	1554	——	≥6 months	HR, DFS	RCT
Swain et al. (73)	——	USA	2,343	1868	——	≥6 months	DFS, OS	RCT
Tham et al. (74)	——	USA	191	115	——	≥6 months	Age, HR, TAM, CR	5
Tiong et al. (75)	2008-2012	Malaysia	102	93	——	≥12 months	Age, TS, TAM	6
Tormey et al. (76)	1972–1982	USA	553	174	43	≥12 months	TAM	RCT
Tormey et al. (77)	1982–1987	USA	533	354	43	≥6 months	DFS, OS	RCT
Turnbull et al. (78)	2005–2010	UK	107	81	43	No definition	Age	5
Vanhuyse et al. (79)	1985–1995	France	130	74	42.9	≥6 months	Age, ER, PR, DFS, OS	8
Vehmanen et al. (80)	1989–2001	Finland	111	79	43	No definition	TAM	8
Xu et al. (81)	2010–2012	China	120	94	——	≥3 months	Age, CR	4
Yoo et al. (12)	2003–2007	Korea	312	180	43	≥12 months	Age, HR, TAM, CR	8
Zhou et al. (82)	2006	China	103	90	44.5	No definition	Age, CR	5
Zhou et al. (83)	2008–2010	China	165	72	42	≥12 months	Age, HR, TAM	5
Zhou et al. (15)	2003–2008	China	170	61	——	≥12 months	Age, TAM	6

CIA, chemotherapy-induced amenorrhea; HR, hormone receptor; ER, estrogen receptor; PR, progesterone receptor; TAM, tamoxifen; CR, chemotherapeutic regimen; TS, tumor stage; DFS, disease-free survival; OS, overall survival.

a The information was not shown in the research articles.

b The study conducted by Sukumvanich, P et al. showed two different definitions of CIA (3 and 6 months) in the research article.

### Quality of Included Studies

The risk of bias of each study included had been evaluated, and the risk of bias in all the studies was within acceptable limits. We used the Cochrane risk-of-bias tool to evaluate the risk of bias in the 22 published RCTs (**Supplementary Figure S1**). Few of the RCTs provided information regarding the blinding method. For the 46 observational studies, the risk of bias was evaluated with a modification of the Newcastle–Ottawa scale (**Supplementary Table S1**). 30 studies were considered to be of high quality.

### Pooled Analyses of Risk Factors

#### Incidence of CIA in Patients With Age ≤40 and Age >40

**Figure 2:**
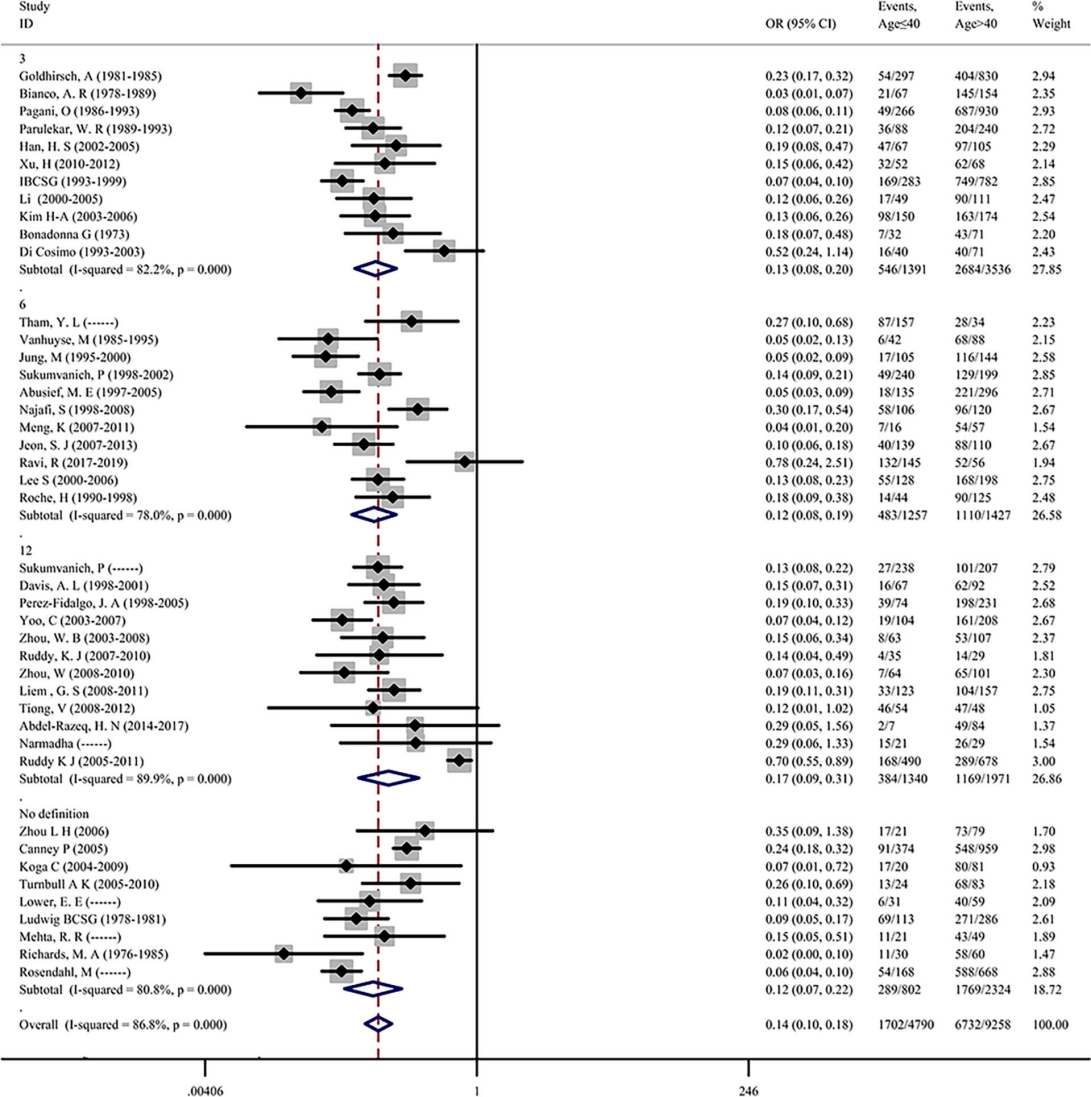
Premenopausal breast cancer patients age ≤40 years versus age >40 years in terms of the incidence of CIA.

More than half of the included studies have reported the effect of age on the occurrence of CIA, indicating that age might be an important predictor of CIA. To first investigate the role of age on the incidence of CIA in premenopausal patients with breast cancer, 43 of the 68 studies containing age information were extracted and the pooled ORs were assessed. The incidence of CIA was 35.53% in patients with an age of ≤40 and 72.72% in patients with an age of >40. The overall pooled ORs of CIA in patients with an age of ≤40 versus an age of >40 was 0.136 (95% CI = 0.104–0.177, p <0.001), indicating that younger patients were less likely to develop CIA. For the detected heterogeneity found among studies (*I* ^2^ = 86.8%), we then divided the studies into 4 subgroups based on the definitions of CIA, and the pooled ORs of the subgroups were assessed (**Figure 2**). We found that the pooled ORs of each subgroup were significant and further found a remarkable decrease in the incidence of CIA for patients with age of ≤40. To explore the study heterogeneity, we investigated the influence of each individual study on the overall meta-analysis summary estimate and found that no study was suspected of excessive influence (**Supplementary Figure S3A**). Significant reporting bias was not detected among studies by Begg’s test (Begg’s p = 0.391, **Supplementary Figure S2A**).

#### Association Between HR Status and CIA Incidence

A total of 14 studies investigated the association between HR status and CIA incidence. The overall pooled OR was 0.611 (95% CI = 0.495–0.753, *p* < 0.001) in patients with HR-negative status versus patients with HR-positive status (**Supplementary Figure S4A**). For the detected heterogeneity among studies (I^2^ = 66.3), a random-effect model was used to assess the pooled ORs, and subgroup analysis was further performed. We found that the OR values were significant in the 3-, 6-, and 12-month groups, except for the “no definition” group including only one trial, suggesting that patients with an HR-negative status are less prone to CIA than patients with an HR-positive status. No publication bias was found during the analysis (Begg’s p = 0.661, **Supplementary Figure S2B**).

Our results showed that the HR status is correlated with the incidence of CIA. We further separately evaluated the effect of ER status and PR status based on information acquired from the studies. A total of 9 studies contained information about ER status and CIA incidence in premenopausal patients with breast cancer. As shown in **Supplementary Figure S4B**, the overall pooled OR was 0.683 (95% CI = 0.518–0.900, p = 0.007) in patients with an ER-negative status versus patients with an ER-positive status. After subgroup analysis, only 6- and 12-month groups contained more than one study, and the OR values were both significant, suggesting that patients with an ER-negative status may have a lower incidence of CIA. No publication bias was found during the analysis (Begg’s p = 0.602, **Supplementary Figure S2C**).

As observed for the group of the relationship between ER status and CIA incidence, we acquired information of PR status from 8 studies, and the pooled OR was assessed. We concluded that patients with a PR-negative status were less prone to CIA than patients with a PR-positive status, as the overall pooled OR was 0.690 (95%CI = 0.495–0.961, p = 0.028) in patients with a PR-negative status versus patients with a PR-positive status (**Supplementary Figure S4C**). No publication bias was found during the analysis (Begg’s p = 0.711, **Supplementary Figure S2D**). Since heterogeneity was found in all 3 analyses above, sensitivity analysis was further performed, and no studies that had a significant impact on the results were found (**Supplementary Figures S3B–D**).

#### Effect of Tamoxifen Use on CIA Incidence

**Figure 3:**
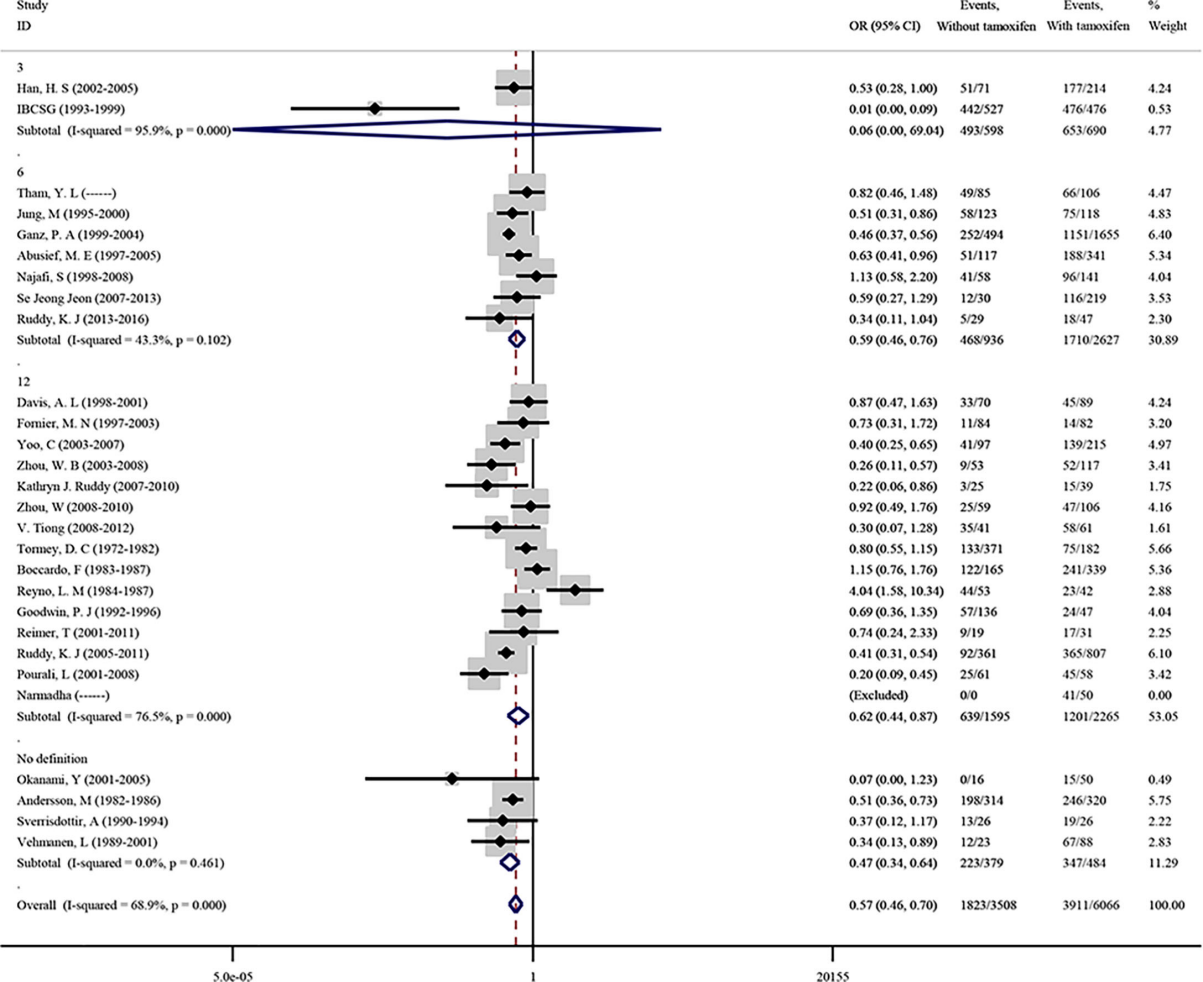
Premenopausal breast cancer patients with or without the administration of tamoxifen on the incidence of CIA.

As a postoperative treatment strategy for premenopausal breast cancer, hormone therapy by tamoxifen (TAM) following chemotherapy for patients with an HR-positive status is recommended (84). To evaluate the effect of tamoxifen on CIA in premenopausal patients with breast cancer, 27 studies were included in this meta-analysis. As shown in **Figure 3**, the overall pooled OR was 0.568 (95%CI = 0.461–0.701, p < 0.001), which showed that the use of tamoxifen can significantly increase the risk of CIA. Subgroup analysis also revealed that tamoxifen could increase the risk of CIA, regardless of the definition of CIA (p < 0.001). Significant reporting bias was not found in the meta-analysis on tamoxifen (Begg’s p = 0.243, **Supplementary Figure S2E**). For the detected heterogeneity found among studies (*I* ^2^ = 68.9%), we then proceeded with the sensitivity analysis, and no study was found to have excessive influence (**Supplementary Figure S3E**).

#### Association Between Chemotherapeutic Regimens and CIA Incidence

**Figure 4:**
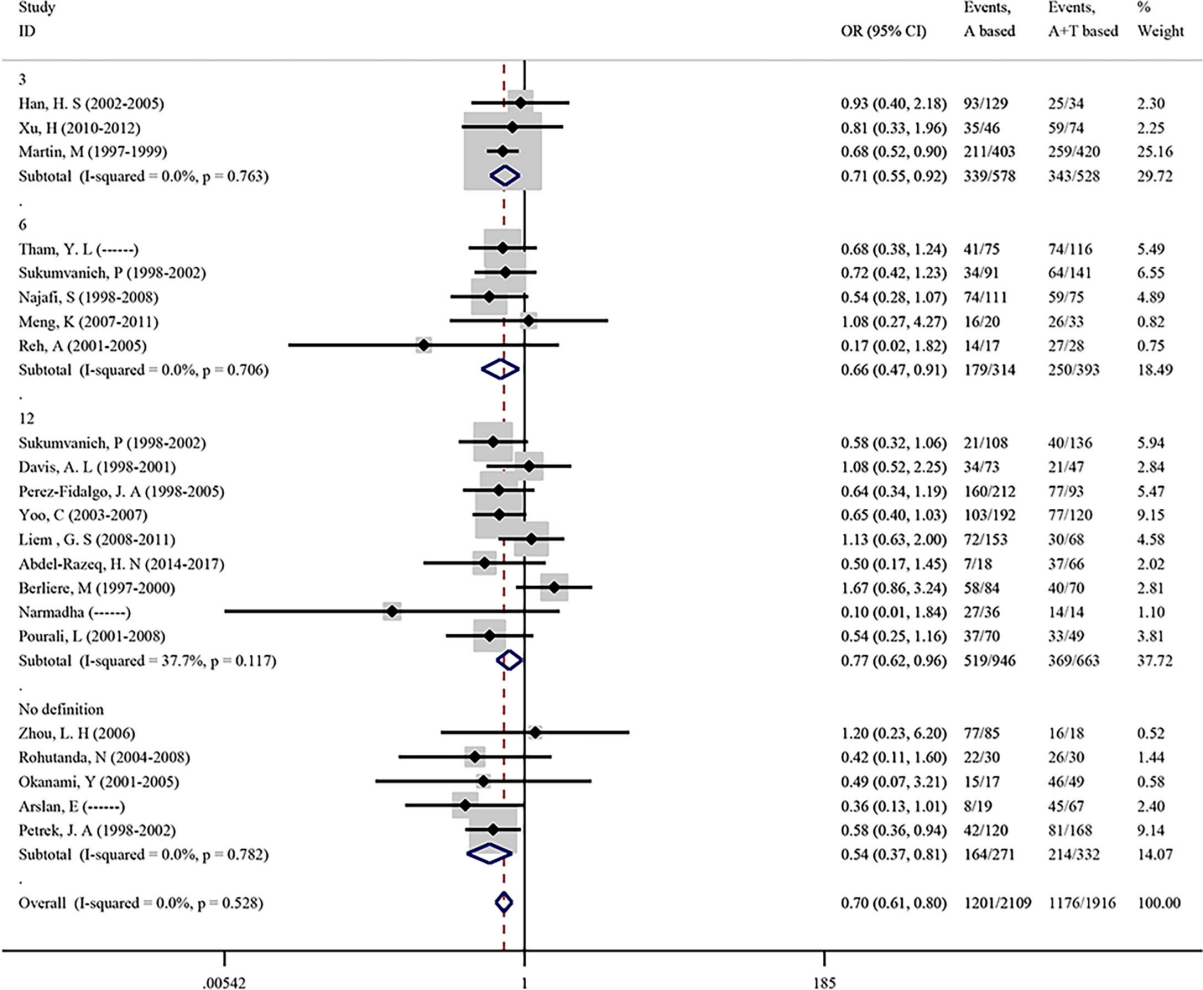
Incidence of CIA based on the application of different chemotherapy regimens.

To evaluate the effect of the two most common chemotherapeutic regimens on CIA incidence, we conducted a meta-analysis focused on the chemotherapy drug which included anthracycline-based (A) and anthracycline-taxane-based (A+T) in 22 studies. As shown in **Figure 4**, the overall pooled OR in anthracycline-based (A) versus anthracycline-taxane-based (A+T) was 0.699 (95% CI = 0.608–0.803, p <0.001), suggesting that taxane can significantly increase the incidence of CIA. No heterogeneity (I^2^ = 0.0%) and publication bias (Begg’s p = 0.236, **Supplementary Figure S2F**) were found in the analysis of chemotherapeutic regimens.

#### Relationship Between Tumor Stage and CIA Incidence

We found that 5 studies involving 733 patients reported the relationship between tumor stage and CIA incidence, with 60.17% of patients developing CIA during stage I/II and 70.77% patients during the III/IV stage. After meta-analysis, the overall pooled OR of CIA in stages I and II versus III and IV was 0.765 (95% CI = 0.512–1.145, p = 0.193), suggesting that there was no correlation between the tumor stage and CIA incidence (**Supplementary Figure S5**). The detected heterogeneity of overall studies was low (*I* ^2^ = 15.9%). The publication bias of this analysis was calculated using Begg’s test, and no significant publication bias was found (Begg’s p = 0.806, **Supplementary Figure S2G**).

### The Prognostic Effect of CIA in Premenopausal Patients With Breast Cancer

**Figure 5:**
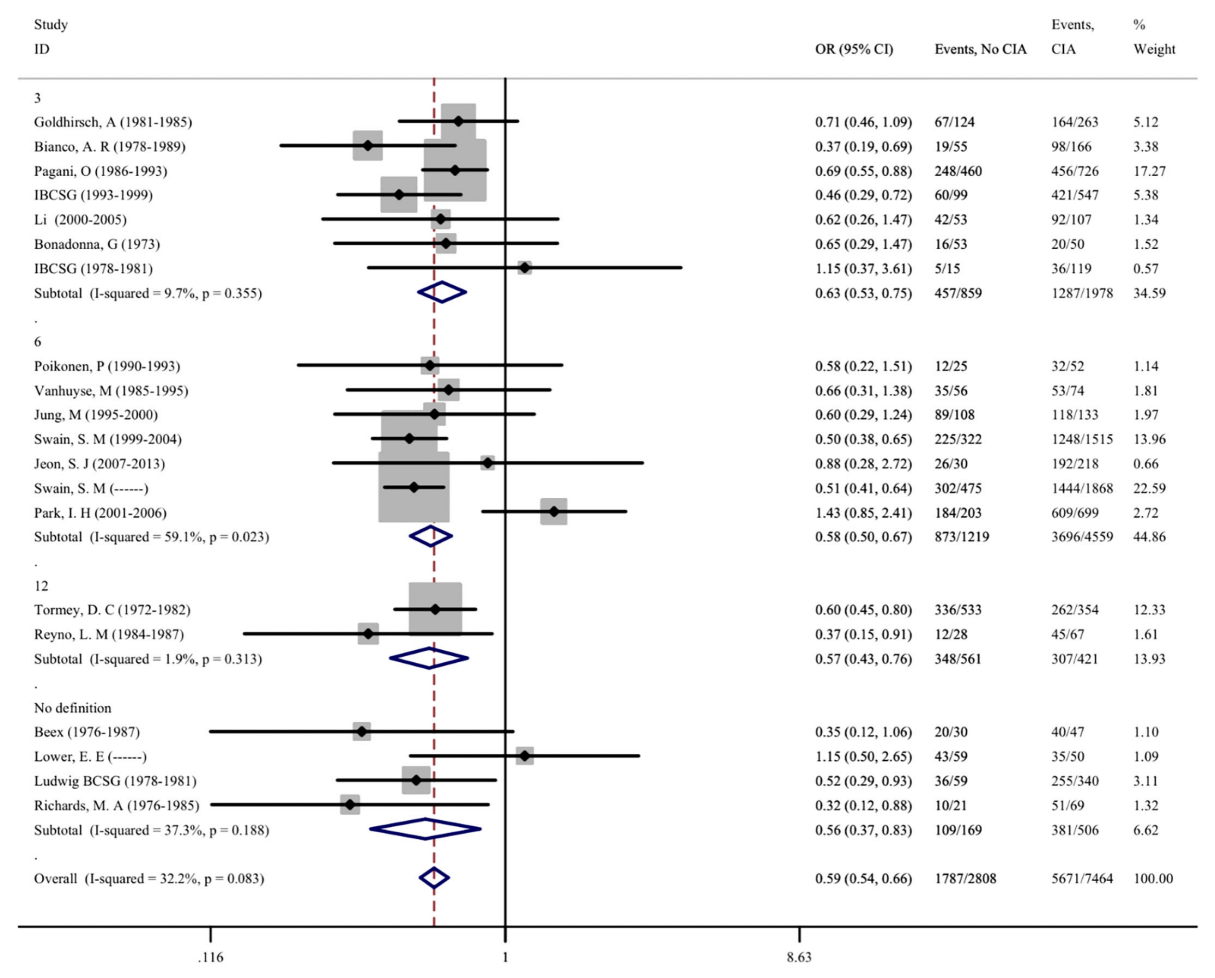
No CIA versus CIA on DFS in premenopausal breast cancer patients.

**Figure 6:**
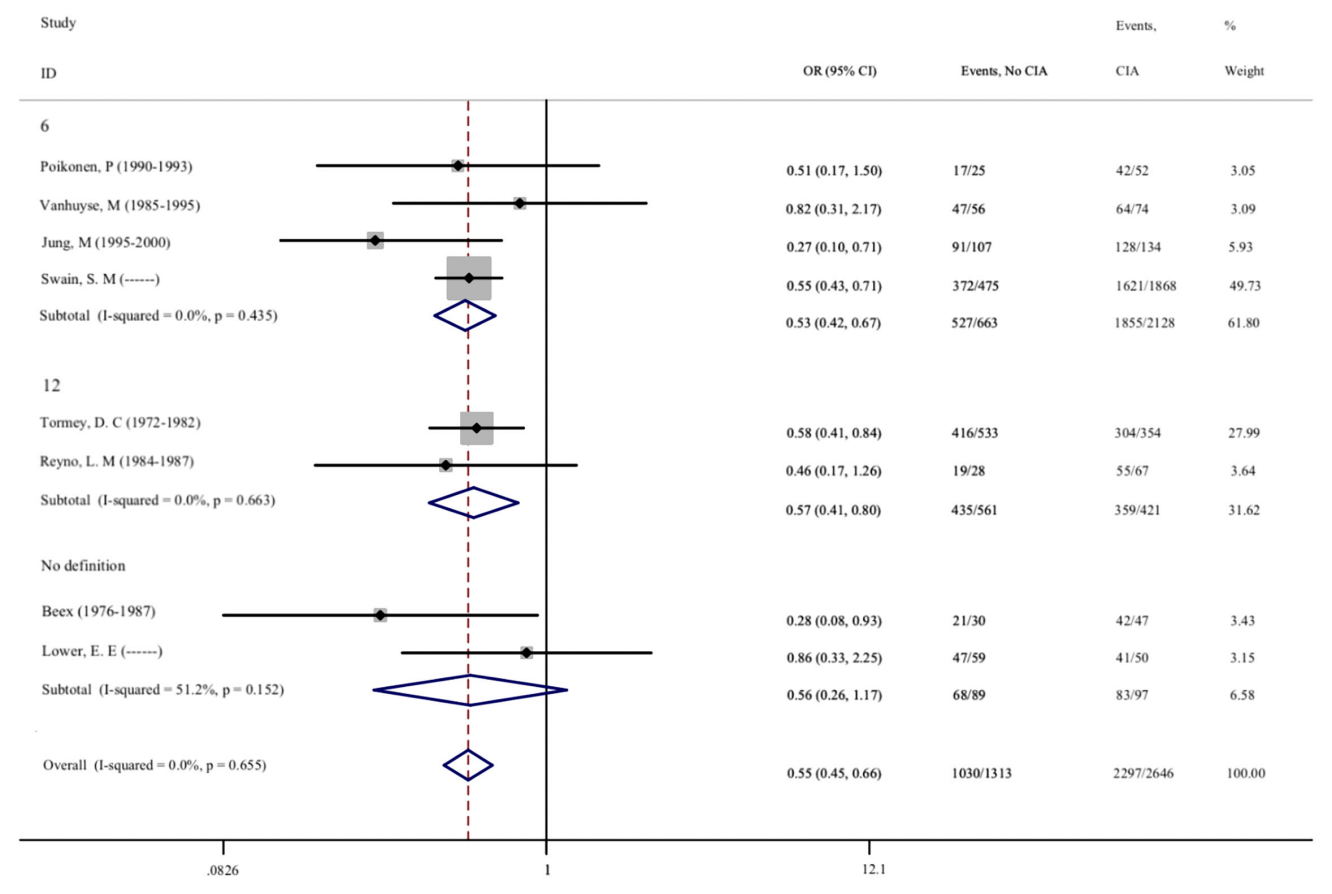
No CIA versus CIA on OS in premenopausal breast cancer patients.

As CIA is one of the most common side effects of adjuvant chemotherapy, we further evaluated the correlation between CIA and disease prognosis in patients. A total of 20 studies involving 11,163 patients were included, and the effects of CIA on the 5-year DFS of patients were evaluated. Moreover, 8 studies that assessed the 5-year OS of patients were included. After meta-analysis, we found that the pooled OR of DFS for premenopausal patients with breast cancer and without CIA was 0.595 (95% CI = 0.537–0.658, *p* < 0.001) compared with patients with CIA, and the OR of OS was 0.547 (95%CI = 0.454–0.660, p <0.001). The same results were also found in different groups after subgroup analysis, indicating that patients who developed CIA after chemotherapy had a significantly better prognosis (**Figures 5**, **6**). The heterogeneity in the analysis of DFS was not significant (I^2^ = 32.2) and no publication bias (Begg’s p = 0.581, **Supplementary Figure S2H**) was found in the analysis of the prognostic value of CIA. No heterogeneity (I^2^ = 0.0%) and publication bias (Begg’s p = 0.386, **Supplementary Figure S2I**) were found in the analysis of OS. We investigated the DFS and OS effect of each individual study on the result by sensitivity analysis to further explore the heterogeneity of the included studies and found that no study was suspected of having a noticeable effect (**Supplementary Figures S3H, I**).

## Discussion

Our meta-analysis provides an updated, more reliable, and comprehensive conclusion on the risk and prognostic effect of CIA in premenopausal women with breast cancer. This meta-analysis will serve as a tool to help doctors in counseling patients on fertility issues. This meta-analysis consisted of 68 studies and 26,585 premenopausal patients with breast cancer, and all the patients are in early-stage breast cancer except 5 patients in 1 study published by Najafi (54). Based on the 68 studies included through inclusion and exclusion criteria, a total of 7 factors that may affect the incidence of CIA were assessed, among which age of patients, HR status, ER status, PR status, use of tamoxifen, and use of anthracycline-based (A)/anthracycline-taxane-based (A+T) regimens were found to be associated with CIA occurrence. No significant correlation was found between tumor stage and CIA incidence. Moreover, the results showed that CIA is associated with favorable disease-free survival (DFS) and overall survival (OS) in premenopausal patients with breast cancer.

Breast cancer in women has now become the most commonly diagnosed cancer worldwide; the data released by the World Health Organization showed that 2.26 million new cases of breast cancer were diagnosed globally in 2020 (1). Adjuvant chemotherapy is necessary for most patients to reduce the risk of recurrence and metastasis, which also prolongs the survival interval (85, 86). As more patients with breast cancer benefit from the use of adjuvant chemotherapy, long-term side effects such as premature ovarian failure presented as CIA have become a major concern (9). Premature ovarian failure is characterized by the suppression of ovarian function, which leads to a menopause-like state in the premenopausal period (87). In previous reports, the quality of life in premenopausal patients with breast cancer and CIA was reported to be impaired because of symptoms associated with premature ovarian dysfunction as well as the other side effects of chemotherapy (20, 88). A significant number of women receive a cancer diagnosis before their age of natural menopause, the most frequent neoplasms included breast cancer, and most of these patients desire to preserve fertility (89); it is important to consider the assessment and management of CIA in the clinical treatment of premenopausal patients with breast cancer. In 2014, Zhao published a meta-analysis (5) that included 46 studies on the risk factors that affected CIA incidence and their prognostic effect. In 7 years, several trials have been reported but no meta-analysis has been reported on this. Although a meta-analysis was published by Zavos (90) in 2016, only 14 studies were included on risk factors associated with CIA, and there is no analysis estimating the prognostic effect of CIA. To further gain a more reliable and comprehensive conclusion, we conducted an updated meta-analysis.

Age was identified as a crucial factor associated with the incidence of CIA in previous studies. Older patients (>40 years) are more likely to develop CIA after adjuvant chemotherapy (68, 91). Moreover, the incidence of CIA is positively correlated with age in more detailed groups in some studies (6, 32). Perez-Fidalgo et al. reported that the risk of amenorrhea increased with the increase of age, and the incidence of CIA in groups with an age of ≤40, 41–45, and >45 was 52.0%, 70.8%, and 95.1%, respectively (6). Furthermore, studies reported that the occurrence time of CIA is negatively correlated with the age of the patients, suggestive of increasing sensitivity to the toxic effect of chemotherapeutic regimens in older women (32, 92). Consistent with previous studies, the present meta-analysis showed that age plays a dominant role in the incidence of CIA. The overall pooled OR for patients with an age of ≤40 versus age of >40 was 0.136, which suggested that CIA is associated with age, and older women are more prone to amenorrhea. Premenopausal patients older than 40 should be informed about the high risk of amenorrhea and its adverse effect on the quality of life.

The effect of HR status on the incidence of CIA has not been well documented in previous studies, and whether the HR status affects the incidence of CIA is debatable. Parulekar et al. reported no difference in CIA risk between two HR status groups, with 73.3% incidence in the receptor-positive group and 74.0% incidence for the receptor-negative group (11). Fornier reported that hormone-positive patients had a significantly increased risk of CIA (37). Results from our meta-analysis showed that patients with a positive hormone status were more prone to develop CIA. The overall OR was 0.611 for HR-negative patients compared with HR-positive patients, which suggested that the HR status can be a potential predictive factor of CIA incidence in premenopausal patients with breast cancer. In addition, our meta-analysis separately analyzed the relationship between the ER or PR status and CIA incidence, and the results were as follows: the overall ORs were 0.683 for ER-negative patients compared with ER-positive patients, and the overall ORs were 0.690 for PR-negative patients compared with PR-positive patients. This means that patients only with an ER- or PR-positive status also have a higher incidence of CIA. Whether there is a direct biological link between HR status and CIA is still unknown.

The role of tamoxifen in the incidence of CIA is debatable. The IBCSG trial 13-93 that involved 1,293 premenopausal patients with breast cancer showed no difference between the use of tamoxifen and CIA incidence (42). However, some other studies have reported that tamoxifen plays an obvious role in the occurrence of ovarian failure. NSABP B-30 consisting of 708 premenopausal patients showed that the use of tamoxifen could significantly increase the incidence of CIA (10). Our meta-analysis evaluated the effect of tamoxifen on CIA incidence and showed a significant increase in the incidence of CIA after the use of tamoxifen, with overall OR = 0.568, p <0.001 for therapy without tamoxifen versus with tamoxifen. Our meta-analysis further confirmed that the use of tamoxifen would increase the risk of CIA. Based on these results, premenopausal patients with breast cancer willing to preserve their fertility should be informed of the potential risks of amenorrhea while prescribing tamoxifen, and alternative treatments should be recommended.

There is no single worldwide standard adjuvant chemotherapeutic regimen in the treatment of breast cancer, and the preferred regimens are variable. Chemotherapy drugs used for the treatment of breast cancer include cyclophosphamide, epirubicin, fluorouracil, docetaxel, and paclitaxel. The most commonly used chemotherapeutic regimens include anthracycline-based (A) and anthracycline-taxane-based (A+T) regimens (17). Because taxanes are usually administered concomitantly or sequentially with A in anthracycline-taxane-based (A+T) regimens, verifying the true effect of taxanes on CIA is difficult. Based on previous studies, we learned that there are discordant results in the effect of the CIA. Some studies reported that the addition of taxane to anthracycline-based regimens would increase the incidence of CIA (52, 54). However, other studies indicated that taxane had no significant effect on the risk of CIA (15, 93).

Our meta-analysis showed that the incidence of CIA in the A+T group is significantly higher than that in the A group (OR = 0.699), which suggests that the addition of taxane to the anthracycline-based regimen could increase the incidence of CIA in premenopausal women with breast cancer. It indicated that taxane should be used cautiously in chemotherapy for premenopausal patients with breast cancer willing to preserve fertility, and ovarian protective medications should be appropriately administered when patients undergo chemotherapy.

To further evaluate the effect of CIA on the prognosis of patients with breast cancer, we performed an analysis to determine the role of CIA on 5-year DFS and OS. The prognostic effect of CIA has been reported by others, although this has not been a consistent conclusion. A 20-year follow-up of women with breast cancer showed no significant difference in both relapse-free and OS between women with CIA and without CIA (34). However, Park reported that CIA is associated with improved 5-year DFS and OS regardless of the treatment (58). Based on our findings, the incidence of CIA could significantly improve the prognosis of patients with breast cancer in their premenopausal age. Just as the SOFT & TEXT (94)demonstrated that GnRH analogues could improve the prognosis of premenopausal patients by inhibiting ovarian function, this may also explain why CIA could significantly improve the prognosis. Considering the effect of fertility issues on patients’ quality of life due to dysfunction of the ovary, more premenopausal patients use GnRH analogues to protect ovary function during chemotherapy; however, due to their effects on menopausal status, patients with the addition of GnRH analogues are excluded from our analysis. A 5-year follow-up of the S0230/POEMS study (95) demonstrated that the patient with goserelin could avoid premature menopause and preserve future fertility, and DFS and OS were not inferior to those used in the chemotherapy group alone. Lambertini et al. conducted a systemic review (96), observing that the use of GnRH analogues has no significant effect on survival and can significantly decrease the premature ovarian insufficiency (POI) rate. It is proved that adding GnRH analogues in premenopausal breast cancer patients could effectively prevent the occurrence of CIA and has no significant effect on prognosis. There are both positive and negative effects of CIA on prognosis and life quality; therefore, more individualized strategies should be carried out in clinical practice.

Our study has some limitations. First, although we tried to minimize heterogeneity by dividing included studies into 4 subgroups based on the definitions of CIA, the meta-analysis includes some results with statistical heterogeneity (I^2^>**50%**) because of high heterogeneity of size, design, method, and therapeutic regimen, which were not explained by our sensitivity analyses. However, in prevalence meta-analysis, heterogeneity was common, which may be because a large number of sample sizes of individual studies have accurate estimates that can lead to statistical heterogeneity (97). Second, although the quality assessment showed that most studies were of high quality, some studies nevertheless had a small sample size, leading to potential bias. Third, most eligible studies were retrospective, and confounding factors may have biased the results. Fourth, the duration of CIA was not available in most studies we included and was therefore not analyzed in the present study. Despite these limitations, this meta-analysis is robust enough to be considered effective and provides valuable and up-to-date information on the risk factors of CIA and the relationship between CIA and prognostic factors.

## Conclusion

To summarize, this meta-analysis of premenopausal patients with breast cancer and CIA showed that age, HR-positive status, ER-positive status, PR-positive status, use of tamoxifen, and anthracycline-taxane-based (A+T) regimens are significantly associated with a higher incidence of CIA, whereas tumor stage showed no significant correlation. Although the occurrence of CIA might induce fertility dysfunction and other syndromes, CIA was found to be an indicator that correlated with a better prognosis of premenopausal patients with breast cancer. Large-scale prospective cohort studies are necessary to further verify the factors associated with CIA incidence and to confirm the effects of prognostic factors. Based on our study, the CIA is a double-edged sword between the quality of life and prognosis of premenopausal patients with breast cancer; both effects should be considered in clinical treatments to perform individualized treatments.

## Data Availability Statement

Publicly available datasets were analyzed in this study. These data can be found here: Cochrane Library, EMBASE, MEDLINE.

## Author Contributions

YL and QY designed and conceived this meta-analysis. YW and JL were engaged in the collection, extraction, and analysis of data. YW and YL were responsible for writing this article. YW and JL conducted the quality assessment and data analysis. YL was responsible for the English language editing. All authors made their own contributions to this paper and agreed to the final version of this paper for submission.

## Funding

This work was supported by the National Key Research and Development Program (No. 2020YFA0712400), Special Foundation for Taishan Scholars (No. ts20190971), National Natural Science Foundation of China (No. 81874119; No. 82072912), Special Support Plan for National High Level Talents (Ten Thousand Talents Program W01020103), National Key Research and Development Program (No. 2018YFC0114705), Foundation from Clinical Research Center of Shandong University (No.2020SDUCRCA015), and Qilu Hospital Clinical New Technology Developing Foundation (No. 2018-7; No. 2019-3).

## Conflict of Interest

The authors declare that the research was conducted in the absence of any commercial or financial relationships that could be construed as a potential conflict of interest.

## Publisher’s Note

All claims expressed in this article are solely those of the authors and do not necessarily represent those of their affiliated organizations, or those of the publisher, the editors and the reviewers. Any product that may be evaluated in this article, or claim that may be made by its manufacturer, is not guaranteed or endorsed by the publisher.

## Supplementary Material

Supplementary Figure 1 The risk of bias in the randomized controlled studies.

Supplementary Figure 2 The publication bias of the meta-analyses included in this study. **(A)** The publication bias of age ≤40 years versus age >40 years in terms of the incidence of CIA. **(B)** The publication bias of patients with HR negativity versus positivity in the incidence of CIA. **(C)** The publication bias of patients with ER negativity versus positivity in the incidence of CIA. **(D)** The publication bias of patients with PR negativity versus positivity in the incidence of CIA. **(E)** The publication bias of patients with or without the usage of tamoxifen on the incidence of CIA. **(F)** The publication bias of different chemotherapy regimens on the incidence of CIA. **(G)** The publication bias of patients with stage I/II verse stage III/IV in terms of the incidence of CIA. **(H)** The publication bias of CIA on DFS in premenopausal breast cancer patients. **(I)** The publication bias of CIA on OS in premenopausal breast cancer patients.

Supplementary Figure 3 The sensitivity analysis of the subgroup of the meta-analysis. **(A)** The sensitivity analysis of age ≤ 40 years versus age >40 years in the terms of incidence of CIA. **(B)** The sensitivity analysis of patients with HR negativity versus positivity in the incidence of CIA. **(C)** The sensitivity analysis of patients with ER negativity versus positivity in the incidence of CIA. **(D)** The sensitivity analysis of patients with PR negativity versus positivity in the incidence of CIA. **(E)** The sensitivity analysis of patients with or without the usage of tamoxifen on the incidence of CIA. **(F)** The sensitivity analysis of different chemotherapy regimens on the incidence of CIA. **(G)** The sensitivity analysis of patients with stage I/II verse stage III/IV in the terms of incidence of CIA. **(H)** The sensitivity analysis of CIA on DFS in premenopausal breast cancer patients. **(I)** The sensitivity analysis of CIA on OS in premenopausal breast cancer patients.

Supplementary Figure 4
**(A)** Premenopausal breast cancer patients with HR negativity versus positivity in terms of the incidence of CIA. **(B)** Premenopausal breast cancer patients with ER negativity versus positivity in terms of the incidence of CIA. **(C)** Premenopausal breast cancer patients with PR negativity versus positivity in terms of the incidence of CIA.

Supplementary Figure 5 Premenopausal breast cancer patients with stage I/II verse stage III/IV in terms of the incidence of CIA.
