# Supplementary figures and images for: Chemotherapy-Induced Amenorrhea and Its Prognostic Significance in Premenopausal Women With Breast Cancer: An Updated Meta-Analysis
Source: Front Oncol. 2022 Apr 5;12:859974. doi: 10.3389/fonc.2022.859974 (PMC9022106; doi:10.3389/fonc.2022.859974)

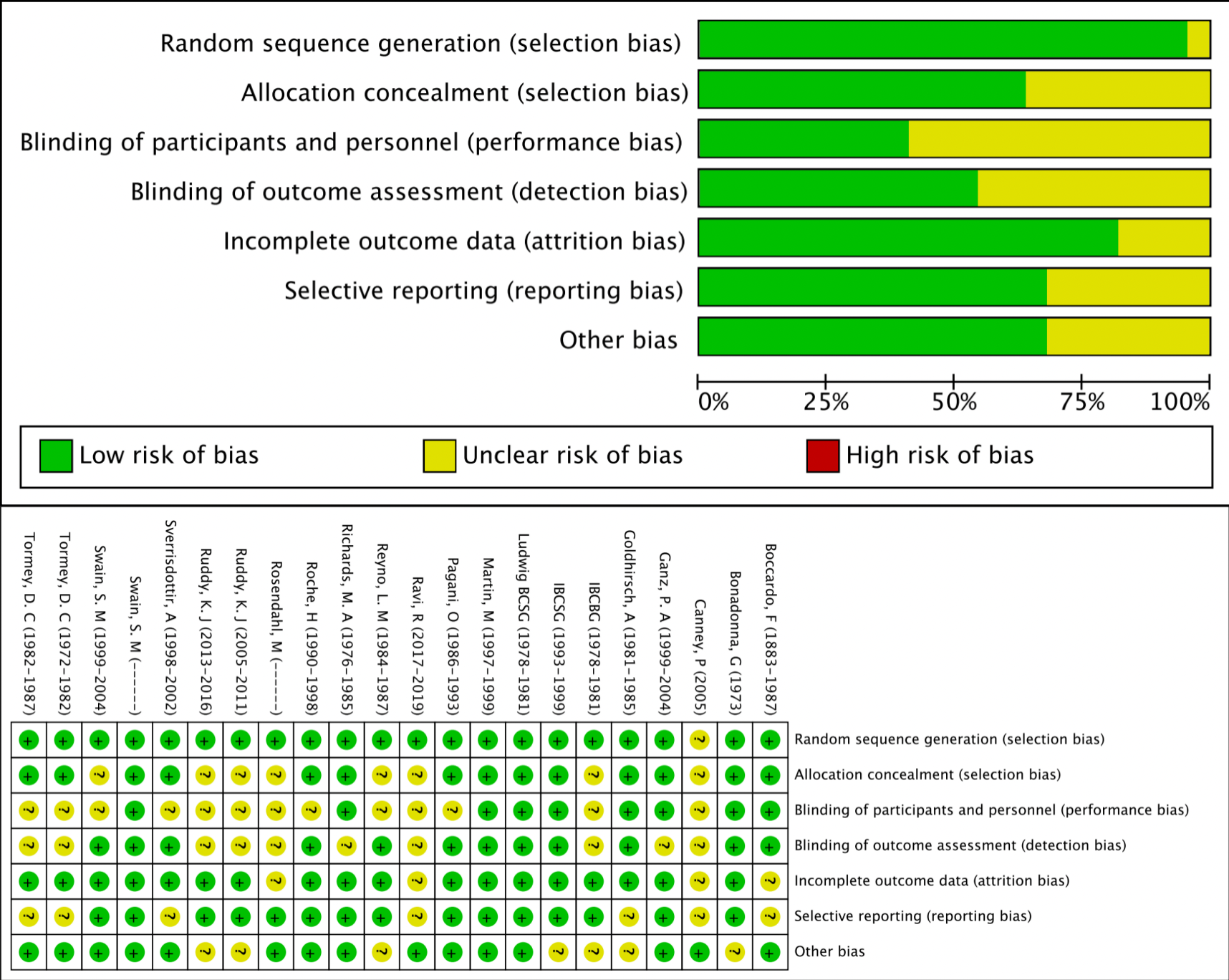

Supplement: Supplementary Figure 1 — The risk of bias in the randomized controlled studies. [file Image_1.tif]

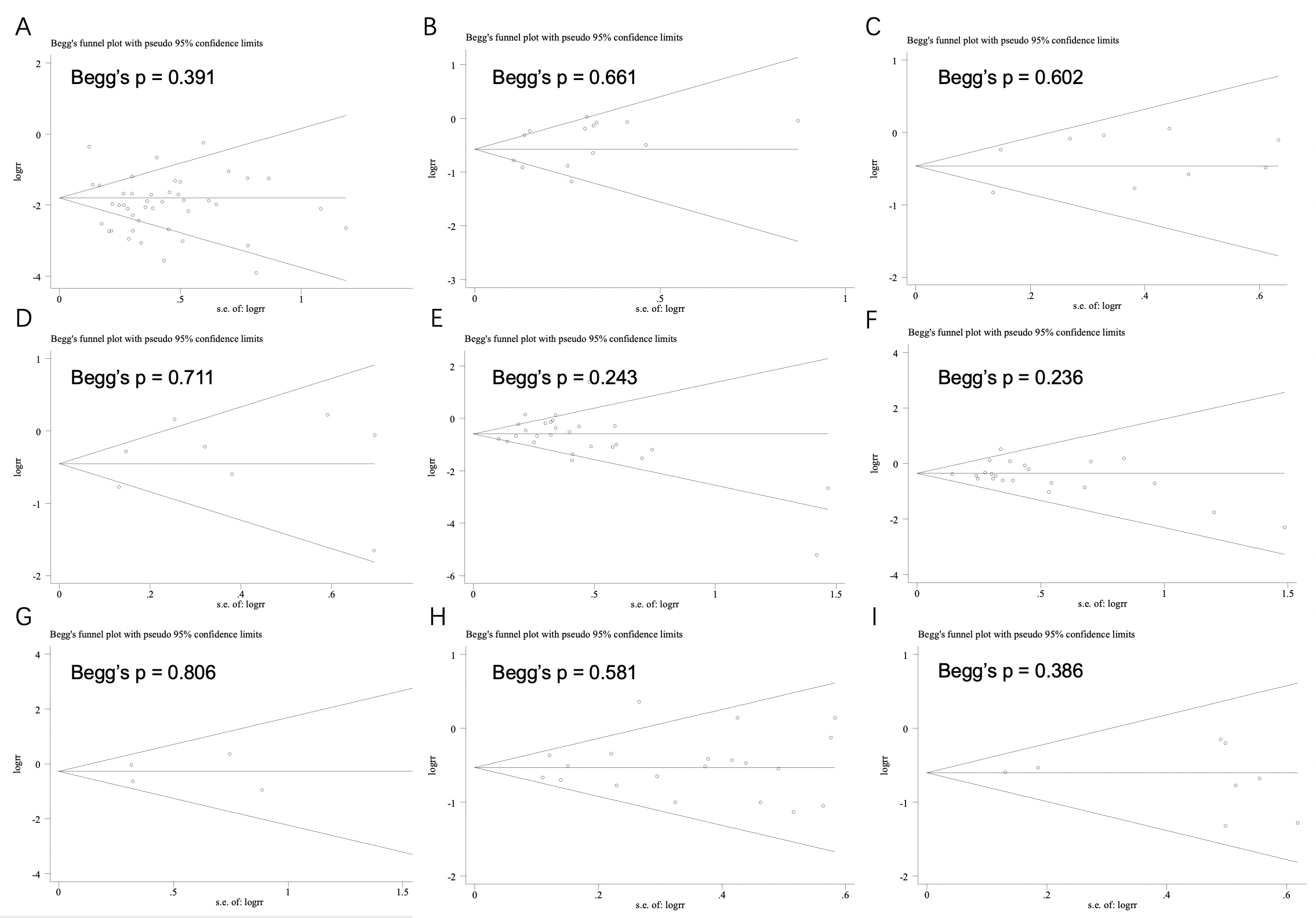

Supplement: Supplementary Figure 2 — The publication bias of the meta-analyses included in this study. (A) The publication bias of age ≤40 years versus age >40 years in terms of the incidence of CIA. (B) The publication bias of patients with HR negativity versus positivity in the incidence of CIA. (C) The publication bias of patients with ER negativity versus positivity in the incidence of CIA. (D) The publication bias of patients with PR negativity versus positivity in the incidence of CIA. (E) The publication bias of patients with or without the usage of tamoxifen on the incidence of CIA. (F) The publication bias of different chemotherapy regimens on the incidence of CIA. (G) The publication bias of patients with stage I/II verse stage III/IV in terms of the incidence of CIA. (H) The publication bias of CIA on DFS in premenopausal breast cancer patients. (I) The publication bias of CIA on OS in premenopausal breast cancer patients. [file Image_2.tif]

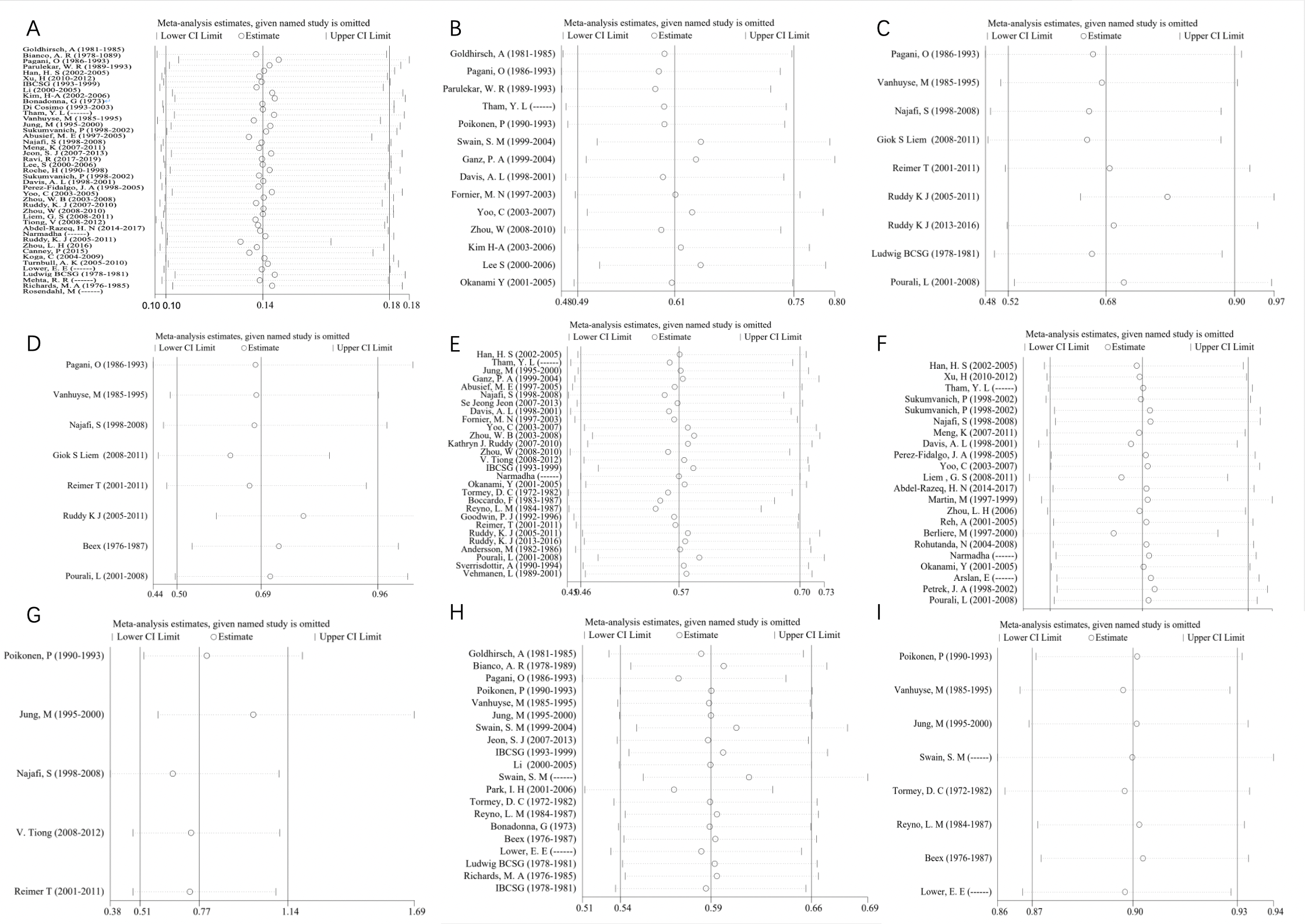

Supplement: Supplementary Figure 3 — The sensitivity analysis of the subgroup of the meta-analysis. (A) The sensitivity analysis of age ≤ 40 years versus age >40 years in the terms of incidence of CIA. (B) The sensitivity analysis of patients with HR negativity versus positivity in the incidence of CIA. (C) The sensitivity analysis of patients with ER negativity versus positivity in the incidence of CIA. (D) The sensitivity analysis of patients with PR negativity versus positivity in the incidence of CIA. (E) The sensitivity analysis of patients with or without the usage of tamoxifen on the incidence of CIA. (F) The sensitivity analysis of different chemotherapy regimens on the incidence of CIA. (G) The sensitivity analysis of patients with stage I/II verse stage III/IV in the terms of incidence of CIA. (H) The sensitivity analysis of CIA on DFS in premenopausal breast cancer patients. (I) The sensitivity analysis of CIA on OS in premenopausal breast cancer patients. [file Image_3.tif]

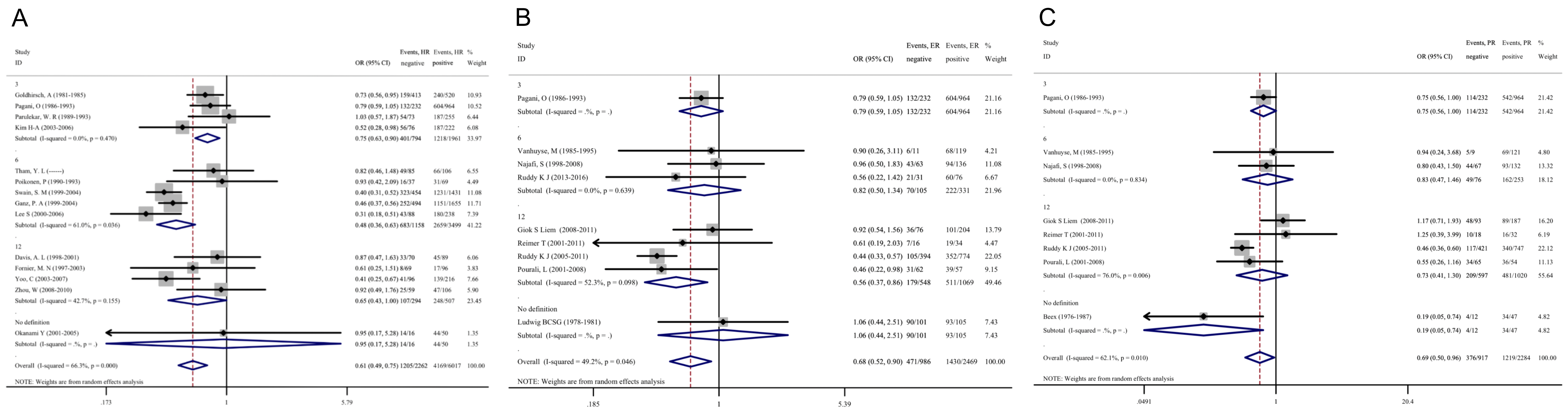

Supplement: Supplementary Figure 4 — (A) Premenopausal breast cancer patients with HR negativity versus positivity in terms of the incidence of CIA. (B) Premenopausal breast cancer patients with ER negativity versus positivity in terms of the incidence of CIA. (C) Premenopausal breast cancer patients with PR negativity versus positivity in terms of the incidence of CIA. [file Image_4.tif]

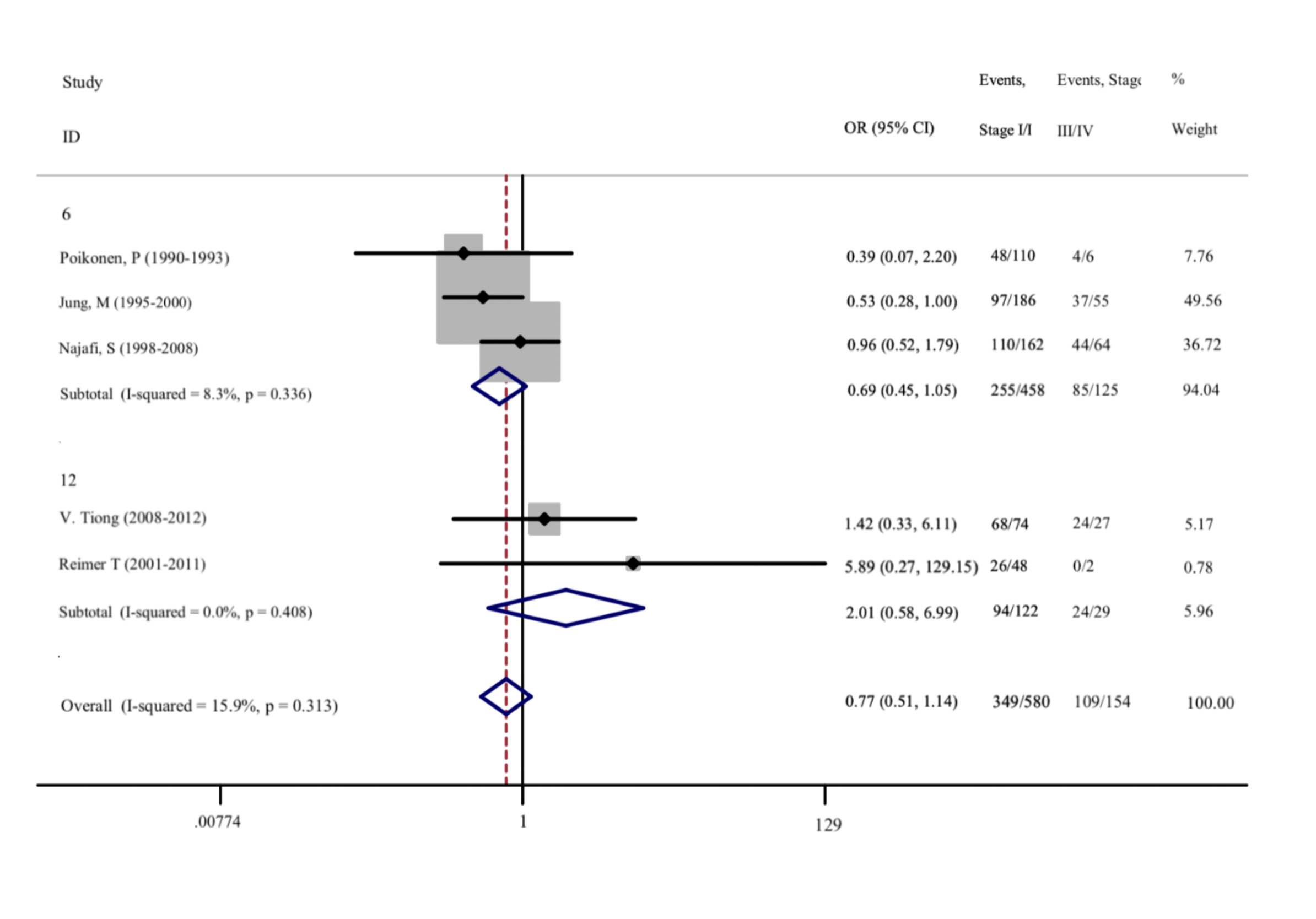

Supplement: Supplementary Figure 5 — Premenopausal breast cancer patients with stage I/II verse stage III/IV in terms of the incidence of CIA. [file Image_5.tif]
